# The effects of emotion and COVID-19 context priming on the size and color of drawings: based on human figure drawings and tree drawings

**DOI:** 10.3389/fpsyg.2023.1278577

**Published:** 2023-12-04

**Authors:** Huijing Cao, Xiaohan Zhang, Xinlei Zhang, Wenhua Yan

**Affiliations:** ^1^School of Psychology and Cognitive Science, East China Normal University, Shanghai, China; ^2^Shanghai Key Laboratory of Mental Health and Psychological Crisis Intervention, East China Normal University, Shanghai, China

**Keywords:** emotion, COVID-19 pandemic, drawing, size, color

## Abstract

**Introduction:**

This study aimed to investigate the effects of emotional themes and the COVID-19 pandemic context priming on the size and color of drawings.

**Methods:**

A 3 (emotion: peacefulness, gratitude, loneliness) × 2 (context: pandemic, regular) mixed design was used and 113 university students in Shanghai were recruited to draw human figures and trees using 10 marker colors.

**Results:**

The size of the drawings depicting loneliness was smaller than that of the those depicting peacefulness and gratitude. Drawings depicting loneliness used more cool and non-preferred colors; drawings depicting gratitude used more warm and preferred colors. Drawings in the pandemic context were larger, which may reflect the more significant threat perceived by individuals. Drawings in the pandemic context used more red colors, showing symbolic meanings such as danger.

**Discussion:**

The drawing size and drawing color are influenced by emotional themes and the pandemic context priming.

## 1 Introduction

The theme of drawing can affect the characteristics of the work, such as its size and color. Several studies have focused on the impact of emotional themes and have found differences in the size and color of drawings depicting different emotional themes (Burkitt et al., 2003a,b, 2004, 2009; Burkitt and Sheppard, 2014). These research primarily recruited children as participants. Most research found that drawings depicting positive emotions are larger than those depicting neutral or negative emotions (Burkitt and Sheppard, 2014). Size can be measured by the height and width of the drawing as well as its area (Burkitt et al., 2003a, 2004, 2009; Burkitt and Barnett, 2006). However, some studies have found that emotional themes do not affect drawing size (Thomas et al., 1989; Jolley, 1995; Strange et al., 2010). It has been argued that the effect of emotional themes is found only when a single object is drawn with other tightly controlled conditions.

In addition to the drawing size, the emotional theme affects the color of the drawing. Controlling for other elements, Jue and Kwon (2013) found that the emotional state of the artist was judged more accurately through colored drawings than after the drawings were processed in black and white, suggesting that color is crucial to the expression of emotions in drawings. Research has found that children use different colors to express a myriad of emotions (Burkitt and Sheppard, 2014). They tend to use colors which they like to represent positive emotions, and colors they do not like to represent negative emotions (Burkitt et al., 2003a,b, 2004, 2009). Furthermore, they tend to associate negative emotions with darker colors such as black and brown (Burkitt and Newell, 2005). Therefore, the characteristics of a drawing can reflect, to some extent, the psychological characteristics of the one who made it.

Several studies have addressed a drawing’s size and color, such as in the case of drawing a human figure (Burkitt and Barnett, 2006), trees (Murayama et al., 2016), and animals (Burkitt et al., 2003a), etc. All of them relate to mental projection techniques. Figure drawing was first used to measure intelligence and is gradually being used for personality analysis, in which the human body is regarded as a medium for self-expression. Individuals’ emotions, psychosexual maturity, anxiety, self-blame, hostility, and many other traits can be inferred from drawings (Moschini, 2005); some researchers have developed indicators for emotional assessments (Koppitz, 1966a,b; Hibbard and Hartman, 1990). Tree drawing is similar to that of a human figure, but is more covert and can lower the defenses of a person drawing it. Tree drawing, introduced by Koch (1949, 1952), uses trees as its only theme. It required the participants to “draw a fruit tree” on blank paper. Simultaneously, Buck (1947, 1948) introduced the House-Tree-Person Technique, which required the participant to draw a house, tree, and person separately on three pieces of blank paper. Considering the cultural differences in the understanding of words, Ji (2007) modified the instruction to “Please draw a tree.”

In early 2020, the COVID-19 pandemic spread rapidly worldwide, becoming a global public health event. The pandemic continues to pose a significant threat to physical and psychological health of humans. Owing to prolonged isolation, fear of infection, increased work stress, sudden lifestyle changes, and deteriorating living conditions, a range of mental health problems have arisen, such as anxiety, depression, sleep disorders, post-traumatic stress disorder, feelings of emptiness, and stress-related disorders (Brooks et al., 2020; Kang et al., 2020; Chen et al., 2021; Saragih et al., 2021). A meta-analysis by Wu et al. (2021) found that the overall combined prevalence of depression, anxiety, distress, and insomnia worldwide was 31.4, 31.9, 41.1, and 37.9%, respectively, during the pandemic, which were significantly higher than previously reported. Thus, a particular contextual factor of the pandemic may also manifest itself in the characteristics of the drawings, thereby influencing their size and color.

This study focused on two typical emotions in the context of COVID-19: loneliness and gratitude. Loneliness is a negative emotion caused by an individual’s perceived unmet social needs (Hawkley and Cacioppo, 2010). To curb the spread of COVID-19, countries have taken measures to reduce human contact by limiting the gathering of people and closing schools and workplaces. These measures have reduced the spread of the coronavirus but have led to widespread social isolation (Landmann and Rohmann, 2022). Consequently, the resulting isolation can be pervasive and devastating (Liu et al., 2020). Studies have shown that more than one-third of the United Kingdom population felt lonely “sometimes or often” during the pandemic (Li and Wang, 2020); and a Chinese survey showed that between March and April 2020, levels of loneliness among the general population increased compared to previous findings in recent years (Sheng et al., 2020).

Combating the negative emotions caused by COVID-19 is currently a hot topic of research. One possible way is to focus on the positive aspects of the pandemic, which cannot only help reduce feelings of isolation, but also give people the strength to overcome such difficulties (Yamaguchi et al., 2020). Gratitude is a positive emotion experienced when an individual is helped and inspired by a willingness to give back (McCullough et al., 2001). The world witnessed many moving moments during the pandemic: healthcare workers being the “heroes in harm’s way” on the front lines, workers toiling day and night to build the Fangcang shelter hospital, strangers helping each other, and so on. These evoke feelings of gratitude. Gratitude and psychological wellbeing are strongly correlated. Individuals accustomed to gratitude experience fewer negative emotions (He et al., 2015). Moreover, gratitude increases positive coping styles and reduces negative coping styles (Hou et al., 2021).

Based on the context of the COVID-19 pandemic, this study examines the effect of emotions on the size and color of drawings. Previous studies have focused on basic emotions (for example, happiness and sadness) in children (Burkitt and Sheppard, 2014). This study recruited university students (who are adults) as participants and focused on two representative complex emotions in the context of the pandemic: loneliness and gratitude; one being a negative emotion and the other a positive. Furthermore, we used peacefulness as a control (in Chinese: “平和”). The word “平和” is common in the Chinese culture, but it is a bit different from “calm” which was used in previous studies, as it actually has some positive meanings. These settings helped test the generalizability of previous findings and has practical significance. Given the above, four main hypotheses were proposed.

Hypothesis 1: Emotional themes may affect drawing size. The drawings depicting loneliness are smaller than those depicting peacefulness, and the drawings depicting gratitude are larger than that of peacefulness.

Hypothesis 2: Emotional themes may affect drawing colors. Drawings depicting loneliness use cooler and disliked colors, whereas drawings depicting gratitude use warmer colors and liked colors.

Hypothesis 3: Pandemic context may affect drawing size. The size of the drawings in the pandemic context is smaller than in the regular situation.

Hypothesis 4: Pandemic context may affect drawing color. In the pandemic context, preferred colors are used less frequently.

## 2 Methods

The study was approved by the University Committee on Human Research Protection, and all participants signed informed consent forms. Data were collected from November 2021 to January 2022, during which the local epidemic in Shanghai was well controlled, with only a few disseminated cases. In the context of regular epidemic prevention and control, people were less affected by the pandemic than during its initial stages. However, people’s lives and work continue to be affected by the local recurrence of COVID-19 cases.

### 2.1 Participants

A total of 113 university students were recruited online and paid to complete the study. None of the participants had a history of psychiatric disease, brain injury, and color blindness or color weakness. All had normal or corrected normal vision in both eyes and were able to perform the drawing tasks successfully. Each participant completed both human figure drawings and tree drawings. The data were filtered separately using the same method, and invalid data were excluded based on the following criteria: (1) incomplete data, not completing all drawings or answering the relevant questions or questionnaires; (2) the emotional rating of the drawing (see “Drawing Questionnaire” section for details) was ≤2. The rating of 1 ~ 2 in a 5-point Likert scale (1 = not at all, 5 = very much) represents that the drawing was not expressing the emotional theme as per the instructions. Eventually, 102 valid data were collected for the human figure drawings, including 59 men and 43 women, with an average age of 21.2 ± 3.85 years. There were 90 valid data for the tree drawings, including 41 men and 49 women, with an average age of 21.3 ± 1.99 years.

### 2.2 Materials

#### 2.2.1 Drawing materials

The drawing materials included a set of 10-color markers (red, black, orange, green, purple, blue, brown, pink, yellow, and gray) and six sheets of blank A4 paper for drawing. The instructions were presented in written form and bound with an A4 paper. The experimenter provided pencils and erasers on request.

#### 2.2.2 Affect grid

The affect grid developed by Russell et al. (1989) was used as a self-rating tool for emotions. It is a 9 × 9 grid with horizontal coordinates indicating valence (pleasure-displeasure) and vertical coordinates indicating arousal (arousal-sleepiness). The participants were asked to mark the appropriate grid according to their emotional state. In the analysis, the markers were converted into two scores for valence and arousal, both of which were scored from −4 to 4. The scoring of the emotion grid is simple and quick; it is used primarily for rapid and repeated measures of immediate emotional states (Russell et al., 1989).

#### 2.2.3 Drawing questionnaire

Drawing questionnaires were used to collect participants’ descriptions of drawings, with open-ended questions about the characteristics of the human figures (or trees) and the surroundings in drawings, as well as a rating of the emotion of drawings. Following is an example: “Please look at the human (tree) you drew again, what is this human’s (this tree’s) rating on peacefulness (loneliness, gratitude)? Please circle the corresponding numbers.” A 5-point scale was used, with 1 = not at all and 5 = very much.

#### 2.2.4 Perceived Risk of COVID-19 Pandemic Scale

The Perceived Risk of COVID-19 Pandemic Scale (PRCPS) was developed by Xi et al. (2020). It comprises nine items divided into three assessment facets: emotional feelings, cognitive judgments, and mental representations of unusual severity. The question items were scored on a 4 ~ 6-point Likert scale. In this study, Cronbach’s α of the scale was 0.819, representing good reliability.

### 2.3 Procedure

A 3 (emotion: peacefulness, gratitude, loneliness) × 2 (context: pandemic, regular) mixed design was used, with emotion as a within-subjects variable and context as a between-subjects variable. Participants were randomly assigned to two groups: the pandemic group and control group.

**Figure 1:**
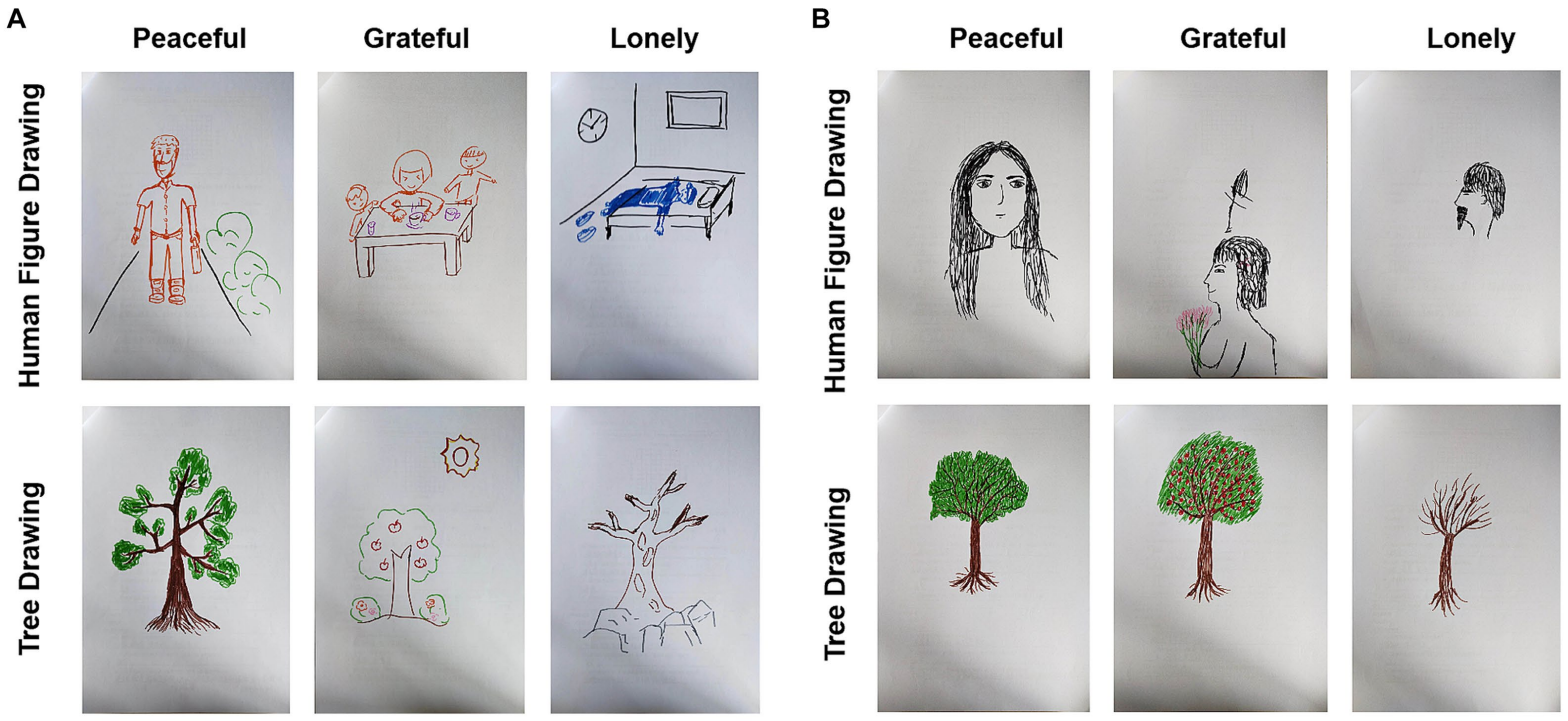
Examples of the drawings. **(A)** Shows the drawings of one participant in the pandemic group. **(B)** Shows the drawings of one participant in the regular group.

The study was administered to groups of 2–10 people, with participants sitting at intervals, and each person being given a set of markers. The experimenter distributed a booklet for drawing instructions and drawing papers. The participants first watched an instructional video on using the grid. After confirming their understanding of the video, they filled in basic personal information (including gender, age, major, grade, and drawing learning experience). Next, the pandemic group read the pandemic priming materials, which consisted of a textual description of the spread of the Omicron variant and two maps of the number of confirmed cases nationwide; the control group did nothing. This was followed by sequential drawings of human figures and trees with three emotional themes, with six drawings in total. The first emotional theme was fixed as a peaceful emotion to balance the order effect. Thereafter, the order of loneliness and gratitude was balanced among the participants. The instruction was as follows: “*Please use the given material to ‘draw a person who is emotionally peaceful (in the pandemic situation)’ on the next blank A4 paper. It can be any person that you want to draw. You are encouraged to use colored pens during the drawing process, and you can use any (one or more) marker. If you need pencils and erasers, you can also ask the experimenters and we will provide them for you.”* Figure 1 shows specific examples of drawings from the participants in the two groups. Time was recorded before and after each drawing, emotional states were assessed with an affect grid, and the drawing questionnaire was filled out after drawing. After completing the drawing of one emotional theme, 21 two-digit addition and subtraction calculations for distraction were performed before proceeding to the next drawing task.

Three days after the drawing tasks were completed, the participants completed the Color Preference Questionnaire and the PRCPS online.

### 2.4 Drawing analysis

The drawing size was measured using height and width. The height of the drawing was defined as the vertical length from the lowest to the highest object, and the width of the drawing was defined as the horizontal length from the leftmost to the rightmost object. The drawings were measured independently by two researchers using a transparent grid plastic ruler, and the average of the two measurements was obtained.

The color of the drawing was scored as 0 or 1, and each of the 10 colors was counted and scored as 1 if the color was used; otherwise, it was scored as 0. The scores for purple, pink, red, orange, and yellow were summed to obtain the number of warm colors used. The scores for green, blue, brown, black, and gray were added to obtain the number of cool colors used. The average preference values for the colors used in each drawing were calculated and defined as the color preference.

## 3 Results

### 3.1 Priming effect check

A paired-sample t-test was conducted on the emotion indicators before and after drawing; the results are shown in Table 1. The valence of emotion significantly increased after drawing of peacefulness and gratitude, it significantly decreased after drawings of loneliness.

**Table 1:** Paired-sample *t-*test for changes in emotion before and after drawing.

Drawing task	Emotional theme	Emotion indicator	Before (*M* ± *SD*)	After (*M* ± *SD*)	*t*	*p*
Human-figure drawing	Peacefulness	Valence	0.38 ± 1.57	1.13 ± 1.42	−4.929	***
		Arousal	0.76 ± 1.61	1.18 ± 1.49	−2.792	0.006
	Loneliness	Valence	−0.17 ± 1.69	−0.91 ± 1.57	4.789	***
		Arousal	0.68 ± 1.81	0.55 ± 1.65	0.731	0.467
	Gratitude	Valence	0.72 ± 1.40	1.25 ± 1.42	−4.283	***
		Arousal	0.81 ± 1.72	1.36 ± 1.57	−4.501	***
Tree drawing	Peacefulness	Valence	0.43 ± 1.54	1.33 ± 1.22	−5.905	***
		Arousal	0.68 ± 1.71	0.82 ± 1.55	−0.940	0.350
	Loneliness	Valence	−0.20 ± 1.33	−0.77 ± 1.43	3.959	***
		Arousal	0.55 ± 1.83	0.49 ± 1.60	0.403	0.688
	Gratitude	Valence	0.49 ± 1.57	1.17 ± 1.48	−3.918	***
		Arousal	0.54 ± 1.82	0.96 ± 1.53	−3.362	0.001

***p < 0.001.

### 3.2 The size of human-figure drawing

A 3 × 2 mixed-design ANOVA was conducted on the height of the human-figure drawings, with the emotional theme as a within-subject factor and pandemic context as a between-subject factor. The results showed a significant main effect of the emotional theme *F*(2,200) = 22.321, *p* < 0.001, partial *η*^2^ = 0.182: The height of the drawings depicting peaceful people (*M* = 10.05) was higher than of those depicting grateful people (*M* = 8.75), *p* = 0.025, and the height of the drawings depicting grateful people were higher than of those depicting lonely people (*M* = 6.52), *p* < 0.001. Neither the main effect of the pandemic context [*F*(1,100) = 0.525, *p* = 0.470, partial *η*^2^ = 0.005] nor the interaction [*F*(2,200) = 0.719, *p* = 0.478, partial *η*^2^ = 0.007] were significant. Considering that the activation of the pandemic context may not be effective for participants with a lower perceived risk of COVID-19, the same analysis was redone with the top 50% of participants scoring on the PRCPS. The results showed a marginally significant main effect of the pandemic context, *F*(1, 51) = 3.896, *p* = 0.054, partial *η*^2^ = 0.071; that is, drawings of the pandemic group (*M* = 9.63) were higher than those of the regular group (*M* = 7.45). However, the interaction effect was insignificant.

Furthermore, the width of the human figure drawings was analyzed similarly. The main effect of the emotional theme was significant, *F*(2,200) = 18.253, *p* < 0.001, partial *η*^2^ = 0.154: the width of the drawings depicting peaceful people (*M* = 5.94) was more than of those depicting grateful people (*M* = 5.23), *p* = 0.048, and the width of the drawings depicting grateful people was more than of those depicting lonely people (*M* = 3.93), *p* < 0.001. The main effect of the pandemic context was not significant, *F*(1,100) = 0.173, *p* = 0.678, partial *η*^2^ = 0.002. The interaction was also not significant, *F*(2,200) = 0.070, *p* = 0.910, partial *η*^2^ = 0.001. The same analysis was conducted again after choosing the top 50% of participants scoring on the PRCPS. A significant main effect of pandemic context was found, *F*(1, 51) = 5.977, *p* = 0.018, partial *η*^2^ = 0.105, with the drawings of the pandemic group (*M* = 5.95) being wider than those of the control group (*M* = 4.38); however, the interaction was still not significant.

### 3.3 The size of tree drawing

A 3 × 2 mixed-design ANOVA was conducted on the height of the tree drawings, with the emotional theme as a within-subject factor and pandemic context as a between-subject factor. The results showed a significant main effect of emotional theme, *F*(2,176) = 30.076, *p* < 0.001, partial *η*^2^ = 0.255: peacefulness trees (*M* = 16.03) were higher than lonely trees (*M* = 11.719), *p* < 0.001; grateful trees (*M* = 15.13) were higher than lonely trees, *p* < 0.001. The main effect of pandemic context was not significant, *F*(1, 88) = 0.153, *p* = 0.696, partial *η*^2^ = 0.002. The interaction was marginally significant, *F*(2,176) = 2.891, *p* = 0.058, partial *η*^2^ = 0.032. Simple effect analysis found that the peacefulness tree (*M* = 16.17) was higher than the grateful tree (*M* = 14.67) in the pandemic context (*p* = 0.057), whereas in the regular context, the peacefulness tree (*M* = 15.90) did not differ from the grateful tree (*M* = 15.59), *p* = 0.696.

Similarly, a 3 × 2 mixed-design ANOVA was conducted on the width of the tree drawings. It revealed a significant main effect for the emotional theme, *F*(2,176) = 31.976, *p* < 0.001, partial *η*^2^ = 0.267: the peacefulness tree (*M* = 11.79) was wider than the lonely tree (*M* = 7.93), *p* < 0.001; the grateful tree (*M* = 10.91) was wider than the lonely tree, *p* < 0.001. The main effect of pandemic context was not significant, *F*(1,88) = 0.472, *p* = 0.494, partial *η*^2^ = 0.005. However, the interaction was significant, *F*(2,176) = 3.313, *p* = 0.039, partial *η*^2^ = 0.036. Simple effect analysis found that the peacefulness tree (*M* = 11.96) was wider than the grateful tree (*M* = 10.55) in the pandemic context, *p* = 0.050, whereas the peacefulness tree in the regular context (*M* = 11.62) did not differ from the gratitude tree (*M* = 11.28), *p* = 0.641. In terms of loneliness, the width of the tree in the pandemic context (*M* = 8.86) was significantly larger than that in the regular context (*M* = 6.99), *p* = 0.040.

### 3.4 The color of human-figure drawing

First, the total number of colors was examined using a 3 × 2 mixed-design ANOVA, with the emotional theme as a within-subject factor and pandemic context as a between-subject factor. It was found that the main and interaction effects were not significant.

Second, a 3 × 2 × 2 mixed-design ANOVA was conducted on the hue of drawings, with emotional theme and color tone as within-subject factors and pandemic context as a between-subject factor. The results showed a significant interaction effect between emotional themes and color tone, *F*(2,200) = 9.395, *p* < 0.001, partial *η*^2^ = 0.086. Simple effect analysis revealed that the number of warm colors (*M* = 1.49) was less than the number of cool colors (*M* = 2.11) in the peacefulness drawings, *p* < 0.001. The number of warm colors (*M* = 1.16) was less than the number of cool colors in the lonely drawings (*M* = 2.13), *p* < 0.001. The number of warm colors (*M* = 1.89) was not significantly different from the number of cool colors (*M* = 1.67) in the gratitude drawings, *p* = 0.064. The interaction between the pandemic context and color tune was not significant, *F* (1,100) = 1.590, *p* = 0.210, partial *η*^2^ = 0.016.

Again, a 3 × 2 mixed-design ANOVA was conducted using each of the 10 colors, with the emotional theme as a within-subject factor and the pandemic context as a between-subject factor. The results revealed that the main effects of emotional themes were significant in the use of red, green, black and gray, as detailed in Table 2. Additionally, there was a significant main effect of the pandemic context, *F*(1,100) = 4.815, *p* = 0.031, partial *η*^2^ = 0.046; red was used significantly more in the pandemic group (*M* = 0.46) than in the non-pandemic group (*M* = 0.33).

**Table 2:** *Post hoc* comparison of the effect of emotion theme on the use of colors in human figure drawing.

Color	*F*	*p*	Partial *η*^2^	*Post hoc* comparison	*p*
Purple	0.510	0.601	0.005		
Pink	1.755	0.176	0.017		
**Red**	**21.703**	***	**0.178**	Gratitude (*M* = 0.62) > peacefulness (*M* = 0.35)	***
				Peacefulness (*M* = 0.35) > loneliness (*M* = 0.23)	0.033
Orange	1.492	0.227	0.015		
Yellow	0.569	0.567	0.006		
**Green**	**5.181**	**0.006**	**0.049**	Peacefulness (*M* = 0.32) > loneliness (*M* = 0.15)	***
				Gratitude (*M* = 0.26) > loneliness (*M* = 0.15)	0.042
Blue	1.755	0.176	0.017		
Brown	1.727	0.181	0.017		
**Black**	**5.046**	**0.007**	**0.048**	Loneliness (*M* = 0.87) > peacefulness (*M* = 0.76)	0.007
				Loneliness (*M* = 0.87) > gratitude (*M* = 0.77)	0.007
**Gray**	**10.390**	***	**0.094**	Loneliness (*M* = 0.57) > peacefulness (*M* = 0.41)	0.003
				Loneliness (*M* = 0.57) > gratitude (*M* = 0.32)	***

***p < 0.001. Bolded values indicate a significant main effect of emotion, allowing for post hoc comparisons.

Finally, a 3 × 2 mixed-design ANOVA was conducted on color preference, with the emotional theme as a within-subject factor and pandemic context as a between-subject factor. The results showed that neither the main effects nor the interaction effects were significant.

### 3.5 The color of tree drawing

A 3 × 2 mixed-design ANOVA was conducted on the total number of colors, with emotional theme as a within-subject factor and pandemic context as a between-subject factor. A significant main effect of the emotional theme was found, *F*(2,176) = 38.188, *p* < 0.001, partial *η*^2^ = 0.303. The number of colors used in drawings of gratitude (*M* = 4.61) was greater than that in drawings of peacefulness (*M* = 4.00), *p* = 0.003, which in turn was greater than the number of colors in drawings of loneliness (*M* = 2.93), *p* < 0.001. The main effect of the pandemic context was not significant, *F*(1, 88) = 0.036, *p* = 0.849, partial *η*^2^ < 0.001; the interaction was also not significant, *F*(2,176) = 0.353, *p* = 0.703, partial *η*^2^ = 0.004.

Next, a 3 × 2 × 2 mixed-design ANOVA was conducted on the hue of drawings, with emotional theme and color tone as within-subject factors, and pandemic context as a between-subject factor. The results showed a significant interaction between the emotional theme and color tone, *F*(2,176) = 11.026, *p* < 0.001, partial *η*^2^ = 0.111. Simple effect analysis revealed that the number of warm colors (*M* = 1.86) was less than the number of cool colors (*M* = 2.14) in drawings of peacefulness, *p* = 0.015, and the number of warm colors (*M* = 1.24) was less than the number of cool colors (*M* = 1.69) in drawings of loneliness, *p* < 0.001. Gratitude drawings had warmer colors (*M* = 2.47) than cool colors (*M* = 2.14), *p* = 0.032. The interaction between epidemic context and color tune was not significant, *F*(1, 88) = 0.008, *p* = 0.931, partial *η*^2^ < 0.001.

Again, a 3 × 2 mixed-design ANOVA was conducted using each of the 10 colors, with the emotional theme as a within-subject factor and the pandemic context as a between-subject factor. The results revealed that the main effects of emotional themes were significant in the use of pink, red, orange, yellow, green, blue, and gray, as detailed in Table 3. None of the main effects of the pandemic context were significant.

**Table 3:** *Post hoc* comparison of the effect of emotion theme on the use of colors in tree drawing.

Color	*F*	*p*	Partial *η*^2^	*Post-hoc* comparison	*p*
Purple	1.591	0.208	0.018		
**Pink**	**6.301**	**0.003**	**0.067**	Peacefulness (*M* = 0.11) > loneliness (*M* = 0.01)	0.006
				Gratitude (*M* = 0.16) > loneliness (*M* = 0.01)	***
**Red**	**42.182**	***	**0.324**	Gratitude (*M* = 0.73) > peacefulness (*M* = 0.42)	***
				Peacefulness (*M* = 0.42) > loneliness (*M* = 0.13)	***
**Orange**	**9.941**	***	**0.102**	Gratitude (*M* = 0.31) > peacefulness (*M* = 0.17)	0.016
				Peacefulness (*M* = 0.17) > loneliness (*M* = 0.08)	0.045
**Yellow**	**5.970**	**0.003**	**0.064**	Gratitude (*M* = 0.41) > loneliness (*M* = 0.19)	***
**Green**	**75.239**	***	**0.461**	Peacefulness (*M* = 0.98) > gratitude (*M* = 0.88)	0.006
				Gratitude (*M* = 0.88) > loneliness (*M* = 0.40)	***
**Blue**	**3.308**	**0.039**	**0.036**	Gratitude (*M* = 0.43) > loneliness (*M* = 0.27)	0.006
Brown	0.400	0.671	0.005		
Black	0.538	0.585	0.006		
**Gray**	**11.513**	***	**0.116**	Loneliness (*M* = 0.48) > peacefulness (*M* = 0.28)	***
				Loneliness (*M* = 0.48) > gratitude (*M* = 0.20)	***

***p < 0.001. Bolded values indicate a significant main effect of emotion, allowing for post hoc comparisons.

Finally, a 3 × 2 mixed-design ANOVA was conducted on color preference, with the emotional theme as a within-subject factor and pandemic context as a between-subject factor. The results showed a significant main effect of emotional theme, *F*(2,176) = 4.142, *p* = 0.020, partial *η*^2^ = 0.045; color use preference was higher in grateful drawings (*M* = 3.45) than in lonely drawings (*M* = 3.29), *p* = 0.011. The main effect of the pandemic context [*F*(1, 88) = 0.001, *p* = 0.970, partial *η*^2^ < 0.001], and the interaction effect [*F*(2,176) = 0.403, *p* = 0.654, partial *η*^2^ = 0.005] were not significant.

## 4 Discussion

### 4.1 The effects of emotional theme and pandemic context on the drawing size

This study found that both emotional themes and pandemic contexts affected the size of drawings. In human-figure drawings, both the height and width showed that peacefulness figures were the largest, grateful figures were the second-largest, and lonely figures were the smallest. Moreover, the size of each figure in the pandemic context is larger than that in the regular context. In the tree drawing, no significant differences were observed between the size of peacefulness and grateful trees, but they were both larger than lonely trees. Interaction effect analysis found that peacefulness trees were larger than grateful trees in the pandemic context.

The emotional theme affected drawing size, supporting Hypothesis 1. However, peacefulness drawings were larger than grateful drawings, which contradicted the hypothesis. This may be because peacefulness is a positive emotion in addition to being a neutral emotion, just like gratitude, although the latter was used as a control. A previous study measured different emotions using an affective grid in groups of children aged 4 and 5 years as well as adults. The results showed that all three groups fell on the positive end of the valence dimension when calm emotions were evaluated (Russell and Paris, 1994). This finding suggests that calm is not the same as a “blank” emotional state, but is a relatively positive emotion. This is supported by the results of the present study: there was a significant increase in valence after drawings of peacefulness, and the increase was greater than drawings of gratitude. Thus, this remains consistent with previous findings that positive emotion drawings are larger than neutral and negative emotion drawings (Burkitt and Sheppard, 2014).

The pandemic context affects drawing size, supporting Hypothesis 3. In human figure drawings, participants with high perceptions of pandemic risk drew larger figures in the pandemic context than in the regular context. In the tree drawings, lonely trees in the pandemic context were larger than those in the regular context. A systematic review of 101 studies of patients’ drawings of their illnesses found that the worse the patients’ perceptions of their health status and future expectations, the larger their drawing size (Broadbent et al., 2019). Likewise, individuals in the pandemic context perceived greater health threats; therefore, they drew larger drawings. A large size can be seen as a psychological adaptation mechanism of “compensation” (Tesser, 1988; Zheng and Peng, 2014). Participants used larger human figures or trees to compensate for their sense of disadvantage under the threat of the pandemic.

### 4.2 The effects of emotional theme and pandemic context on the drawing color

This study found that emotional themes affected drawing color. Drawings of loneliness using more cool and disliked colors, and drawings of gratitude using more warm and liked colors, which supports Hypothesis 2. Furthermore, in terms of the total number of colors, the least number of colors was used in lonely drawings. Previous research has indicated that the use of fewer colors may reflect depressive states (Jue and Kwon, 2013). Drawing lonely objects might have triggered the participants’ own lonely mood, thereby prompting use of fewer colors.

Regarding specific colors, red and yellow were used more often in gratitude drawings, green was used more often in peacefulness drawings, and black and gray were used more often in lonely drawings. Red may evoke either positive or negative feelings (Elliot and Maier, 2012), depending on the context. Red is associated with positive interpersonal associations, such as conveying strength and confidence (Burkitt and Sheppard, 2014), and is revered by feudal imperial power in Chinese culture. Currently, red marks an essential color for celebratory occasions, such as weddings and festivals (Feng, 2018). A lexical study found that the metaphorical associations of “positive-red” and “negative-black” existed in Chinese participants’ perceptions (Zhou, 2012). Similarly, yellow is often associated with positive concepts, such as sunlight and brightness (Burkitt and Sheppard, 2014), and is used more often in drawings of positive emotions. Green implies vitality, peacefulness, and nature. A color metaphor study found that green is associated with positive emotions (Ma, 2012). Black and gray were most often used in lonely drawings, which provided a sense of isolation and desolation. Previous research has found that black is used more frequently in negative human figure drawings (Burkitt et al., 2009).

The pandemic context affected the color of drawings, thus supporting Hypothesis 4. This is demonstrated by the fact that human-figure drawings used a red color in the pandemic context than in the regular context. Red reflects a negative connotation, associated with violence, anger, danger, and other negative feelings. In this study, the content of drawings in the pandemic situation involved concepts related to the symbolic meaning of red, such as life threat and danger. Therefore, red was used more frequently in these drawings. The time of data collection was during the normalization of pandemic prevention and control, when people were less affected by the pandemic; however, the potential psychological influence initiated by the pandemic context could be found, which in turn was expressed in the characteristics of drawings.

### 4.3 Limitations and future directions

**Figure 2:**
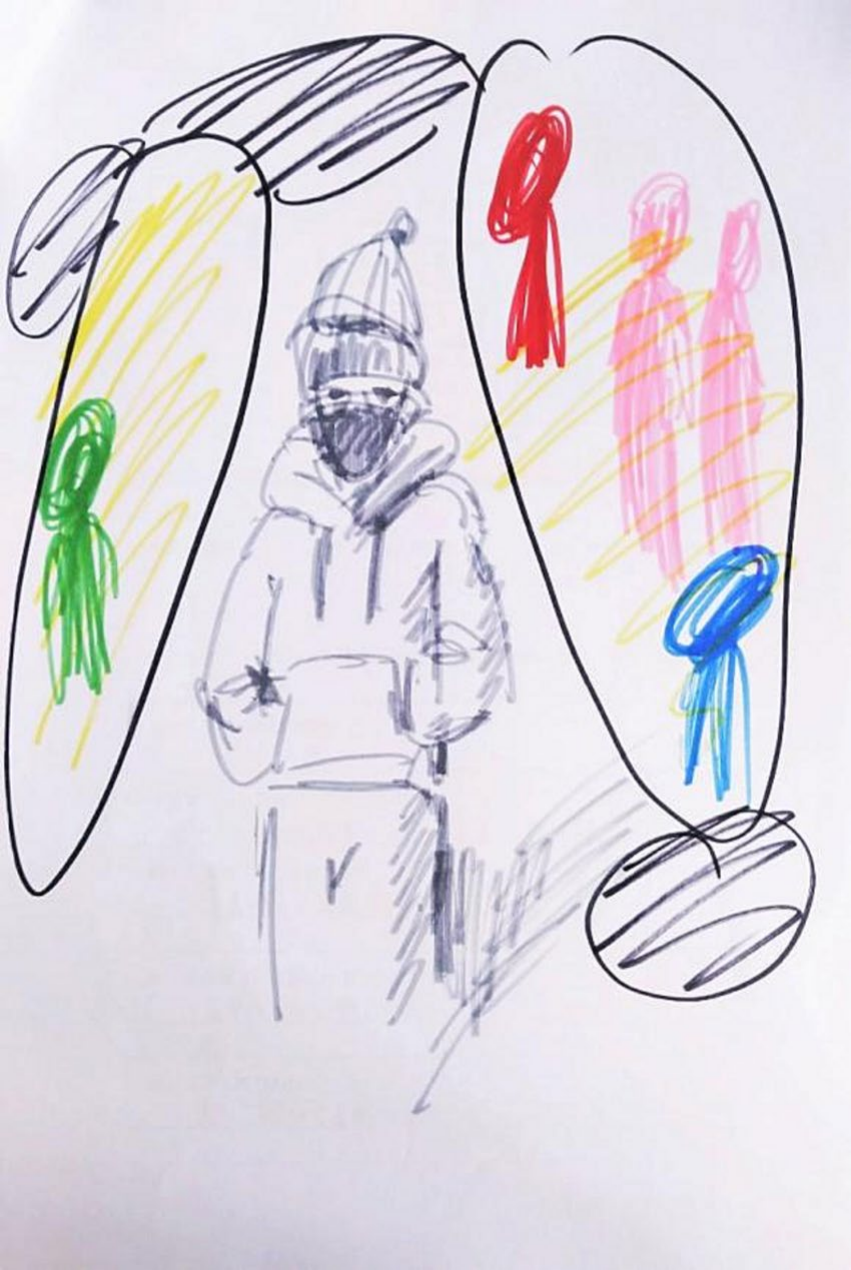
Drawing of a lonely person. This figure shows a drawing depicting a lonely person, which used bright colors to depict the lively environment and used gray to represent the lonely character.

This study examined the effects of emotional themes and pandemic contexts on pictorial characteristics based on both human figure and tree drawings. The results of the two types of drawings were not entirely consistent owing to the special characteristics of the contents. For example, differences in the use of preferred colors were found in tree drawings but not in human-figure drawings. This is probably because the tasks in previous studies mostly required selecting only one color, and the figures did not contain facial expressions or clothing details. The drawing tasks in this study were more liberal and included contour lines, facial features, hair, and many appendages, such as streets and buildings, thereby making the use of color more complex. Moreover, many participants would use contrast to express emotions, such as using multiple bright colors to depict the lively environment, while using gray to represent lonely characters (see Figure 2 for an example). Researchers compared color-in tasks (using one color to color in the outline of a nice/nasty human) with a production task (drawing humans with positive/negative emotional feelings without a limit on the number of colors used) and found an effect of color use preference in the color-in task but not in the multi-colored production task (Crawford et al., 2012). Future research could develop better statistical indicators of colors in drawings, such as separate analyses of colors used in humans or the environment, to better reflect color use in drawings.

## 5 Conclusion

The drawing size is influenced by emotional themes and the pandemic context. The size of the loneliness drawings was smaller than that of the peacefulness and gratitude drawings, verifying that the size of positive emotion drawings was larger than that of negative ones. The drawing size in the pandemic context was larger than that in the regular context, reflecting that individuals perceived a greater health threat in the pandemic context and used compensatory psychological adaptation mechanisms to offset their sense of psychological inferiority.

Drawing color is influenced by emotional themes and the pandemic context. Drawings of loneliness used more cool colors and non-preferred colors, whereas drawings of gratitude used warm colors and preferred colors. Regarding specific color use, gratitude drawings contained more reds and yellows, peacefulness drawings contained more greens, and lonely drawings contained more blacks and grays, which are linked to the color metaphor. Red was used more in drawings of the pandemic context, representing symbolic meanings, such as life threat and danger.

## Data availability statement

The raw data supporting the conclusions of this article will be made available by the authors, without undue reservation.

## Ethics statement

The studies involving humans were approved by University Committee on Human Research Protection of East China Normal University. The studies were conducted in accordance with the local legislation and institutional requirements. The participants provided their written informed consent to participate in this study.

## Author contributions

HC: Conceptualization, Data curation, Formal analysis, Methodology, Writing – original draft. XhZ: Conceptualization, Data curation, Formal analysis, Methodology, Writing – original draft. XlZ: Formal analysis, Writing – review & editing. WY: Conceptualization, Supervision, Writing – review & editing.
